# Evaluation of the pharmacological effects and exploration of the mechanism of traditional Chinese medicine preparation Ciwujia tablets in treating insomnia based on ethology, energy metabolism, and urine metabolomic approaches

**DOI:** 10.3389/fphar.2022.1009668

**Published:** 2022-12-05

**Authors:** Hongda Liu, Le Yang, Chunlei Wan, Zhineng Li, Guangli Yan, Ying Han, Hui Sun, Xijun Wang

**Affiliations:** ^1^ National Chinmedomics Research Center, National TCM Key Laboratory of Serum Pharmacochemistry, Metabolomics Laboratory, Department of Pharmaceutical Analysis, Heilongjiang University of Chinese Medicine, Harbin, China; ^2^ State Key Laboratory of Dampness Syndrome of Chinese Medicine, The Second Affiliated Hospital of Guangzhou, University of Chinese Medicine, Guangzhou, China; ^3^ State Key Laboratory of Quality Research in Chinese Medicine, Macau University of Science and Technology, Macao, China

**Keywords:** Ciwujia tablet, behavioral, energy metabolism, metabolomics, potential biomarkers, tryptophan metabolism, pharmacological effects

## Abstract

Ciwujia Tablets (CWT) are produced by concentrating and drying the extract solution of the dried rhizome of *Eleutherococcus senticosus* (Rupr. & Maxim.) Maxim [Araliaceae; *E. senticosus* radix et rhizoma]. Besides, CWT is included in the 2020 edition of Chinese Pharmacopoeia and is widely used in the treatment of insomnia. It mainly contains eleutheroside B, eleutheroside E, isofraxidin, eleutheroside C, ciwujiatone, and chlorogenic acid, as well as other chemical components. Although the clinical efficacy of CWT in treating insomnia has been confirmed, its functions and pharmacological effects have not been systematically evaluated and its mechanism of action in the treatment of insomnia remains unclear. Therefore, in this study, behavioral, energy metabolism, and metabonomics methods were applied to systematically evaluate the effect of CWT on insomnia. Additionally, urine metabonomics based on UPLC-Q-TOF-MS/MS were utilized to identify potential endogenous biomarkers of insomnia, detect the various changes before and after CWT treatment, explore the metabolic pathway and potential target of CWT, and reveal its pharmacological mechanism. Results revealed that CWT increased inhibitory neurotransmitter (5-HT and GABA) content and reduced the content of excitatory neurotransmitters (DA and NE). Moreover, CWT enhanced autonomous behavioral activity, stabilized emotions, and promoted the return of daily basic metabolic indexes of insomniac rats to normal levels. The urine metabolomics experiment identified 28 potential endogenous biomarkers, such as allysine, 7,8-dihydroneopterin, 5-phosphonooxy-L-lysine, and N-acetylserotonin. After CWT treatment, the content of 22 biomarkers returned to normal levels. The representative markers included N-acetylserotonin, serotonin, N-methyltryptamine, and 6-hydroxymelatonin. Additionally, the metabolic pathways in rats were significantly reversed, such as tryptophan metabolism, folate biosynthesis, phenylalanine metabolism, and tyrosine metabolism. Ultimately, it is concluded that CWT regulated tryptophan metabolism, folate biosynthesis, phenylalanine metabolism, and other metabolic levels in the body. This drug has been confirmed to be effective in the treatment of insomnia by regulating the content of serotonin, 6-hydroxymelatonin, N-acetylserotonin, and N-methyltryptamine to a stable and normal level in tryptophan metabolism.

## 1 Introduction

As a common disease of the central nervous system, chronic or intermittent insomnia is characterized by difficulty in falling asleep or maintaining sleep (Dopheide, 2020; Ai et al., 2021). According to some research, the prevalence of insomnia is about 10%–20%, and about 50% of these patients suffer from chronic insomnia. Irritability and fatigue are common complications of this condition (Buysse, 2013). Additionally, insomnia is often accompanied by mental or physical ailments (Sutton, 2021). It usually induces autonomic nervous system disorders, increases sympathetic activity, leads to brain tension, and increases energy consumption (Grimaldi et al., 2021). Furthermore, insomnia is also a risk factor for other diseases, such as cardiovascular diseases, gastric ulcers, diabetes, and cognitive impairment (Javaheri and Redline, 2017; Tobaldini et al., 2017). It seriously affects patients’ health and quality of life and imposes a huge burden on the healthcare system and society. At present, benzodiazepines are the standard drugs for the treatment of insomnia. However, it is still necessary to identify an alternative due to their side effects. In recent years, prescriptions using traditional Chinese medicine (TCM) have been increasingly applied to the treatment of insomnia worldwide (Singh and Zhao, 2017; Ni et al., 2019).

As a traditional Chinese medicine preparation, Ciwujia Tablets (CWT) are made from the extract of *E. senticosus* (Rupr. & Maxim.) Maxim [Araliaceae; *E. senticosus* radix et rhizoma]. The plant is mainly distributed in China, North Korea, Japan, Russia, and other regions (Jia et al., 2021). According to Chinese Pharmacopoeia, CWT is manufactured by mixing the dried powder of *E. senticosus* with water for decoction, then the extract is concentrated and dried to produce tablets. Through this preparation method, every 3 kg of dried *E. senticosus* can be made into 1,000 Ciwujia tablets, with each tablet containing 0.25 g of extract. These tablets can also be sugar-coated by adding appropriate amounts of excipients (Chinese Pharmacopoeia Commission., 2020). Research shows that CWT mainly contains eleutheroside B, eleutheroside E, isofraxidin, eleutheroside C, ciwujiatone, and chlorogenic acid, as well as other chemical components, especially eleutheroside B, eleutheroside E and isofraxidin can be used as characteristic components of CWT to evaluate their quality. (Liu et al., 2022). It has been demonstrated that *E. senticosus* extract can be used in the treatment of neurodegenerative diseases. Besides, it can be used to regulate autonomic nerve function (Miyazaki et al., 2018) and protect dopamine neurons (Liu et al., 2012). In clinical practice, CWT is primarily used in the treatment of insomnia. Crucially, this drug is generally safe and causes fewer side effects under normal dosages than other drugs (Jia et al., 2021). Although several beneficial effects have been established in previous clinical observations concerning CWT, the curative effect of CWT has not been comprehensively explored. Moreover, the overall pharmacological effects of CWT in the treatment of insomnia have not been systematically evaluated and few relevant studies have been conducted. Furthermore, the mechanism of CWT in the treatment of insomnia is not yet fully understood. Therefore, it is urgent to clarify these issues, to offer a guiding role in the clinical medication of CWT and also provide useful evidence for the pathogenesis and treatment strategies related to insomnia.

The energy consumption rate, water exhalation rate, respiratory entropy, oxygen consumption rate, and other indexes of basic metabolism can be employed to evaluate the pharmacological effects of drugs. These indexes macroscopically characterize the influence of drug treatment on human or animal function. Moreover, metabonomics can be utilized to monitor the changes in small molecule metabolites in the body, reflect the overall functional changes in the body at the metabolic phenotype level (Newgard, 2017), identify disease-related biomarkers, and explore the development mechanism of diseases (Wang et al., 2020). In recent years, metabonomics has become a useful tool for revealing the pathogenesis of diseases and the potential target of drug pharmacological effects (Rinschen et al., 2019; Hu et al., 2020). This study was performed based on behavior, energy metabolism, and metabolomics, in an attempt to evaluate the overall biological effect of CWT on insomnia model rats. Based on the urine metabolomics study and with the assistance of UPLC-Q-TOF-MS/MS technology, potential endogenous biomarkers in insomniac rats were screened by multivariate analysis and identified by a metabolite database. Additionally, the biomarker content before and after treatment was compared, to find the target and pathway of CWT in the treatment of insomnia rats. The reliability and biological significance of the experimental results were verified according to the correlations among the pharmacological effect evaluation indexes, targets, and metabolic pathways. In this study, multiple indexes were applied to comprehensively evaluate the curative effect of CWT and the mechanism of CWT was investigated in combination with urine metabolomics. These findings contribute to further understanding and clarifying the mechanism of the pharmacological effects of TCM.

## 2 Materials and methods

### 2.1 Instruments

In this study, the instruments used included Acquity™ UPLC liquid chromatography (Waters, United States), Synapt™ G2-Si mass spectrometry (Waters, United States), Masslynx v4.1 workstation (Waters, United States), PRO-MRRM-8 small animal energy metabolism monitoring system (Sable Systems International, United States), KQ-250 DB ultrasonic cleaner (Kunshan Ultrasonic Instrument Co., Ltd., China), Sorvall ST 16R table centrifuge (Thermo Scientific, United States), and Thermo Scientific 995 ultra-low temperature refrigerator (Thermo Scientific, United States).

### 2.2 Reagents and materials

Ciwujia Tablets (batch no: 20190701) were purchased from Heilongjiang Wusulijiang Pharmaceutical Co., Ltd. (Harbin, China). Diazepam tablets (batch no: 190405) were obtained from Shandong Xinyi Pharmaceutical Co., Ltd., (Dezhou, China). P-chlorophenylalanine (PCPA) was procured from Shanghai Macklin Biochemical Co., Ltd., (Shanghai, China). PBS phosphate buffer (0.01 mol/L, pH 7.4) was purchased from Shanghai Yuanye Biotechnology Co., Ltd., (Shanghai, China). Normal saline solution (0.9% NaCl) was acquired from Harbin Sanlian Pharmaceutical Co., Ltd., (Harbin, China). MS-grade acetonitrile and methanol were purchased from Thermo Fisher Scientific (Waltham, Ma, United States). Distilled water was obtained from Guangzhou Watson’s Food and Beverage Co., Ltd., (Guangzhou, China). Rat brain tissue 5-HT, GABA, DA, and NE ELISA kits were procured from Nanjing Jiancheng Bioengineering Institute (Nanjing, China).

### 2.3 Animal handling

#### 2.3.1 Animal grouping and preparation of the insomnia model

Male Sprague Dawley (SD) rats (weight 220 ± 20 g) were purchased from Liaoning Changsheng Biotechnology Co., Ltd., (Liaoning, China) and fed adaptively under appropriate conditions for 1 week (temperature 25°C ± 2°C, humidity 50% ± 10%, 12-h light/dark cycle). The activity patterns of the rats were observed in the last 3 days. The SD rats with obvious circadian rhythms, normal food intake and water consumption, and no sharp increase or decrease in body weight were included in the experiment. A total of 80 rats were randomly divided into the control group (C; *n* = 20) and the model group (M; *n* = 60). The group M rats were intraperitoneally injected with 3% PCPA suspension (400 mg/kg) at 9 a.m. every day for four consecutive days, while those in group C were injected with the same amount of normal saline (Si et al., 2020; Yu et al., 2022). After model establishment, ten rats were selected from the C group and the M group, respectively, for model evaluation and metabolomics studies. The remaining M group rats were randomly divided into five groups: the model group (M; *n* = 10), diazepam treatment group (D; *n* = 10), CWT low-dose group (CWT-L; *n* = 10), CWT medium-dose group (CWT-M; *n* = 10), and CWT high-dose group (CWT-H; *n* = 10). The next step of treatment, pharmacological effects evaluation, and metabonomics study were carried out on these five groups of rats and the remaining ten rats in the control group.

#### 2.3.2 Animal therapeutic schedule

**FIGURE 1:**
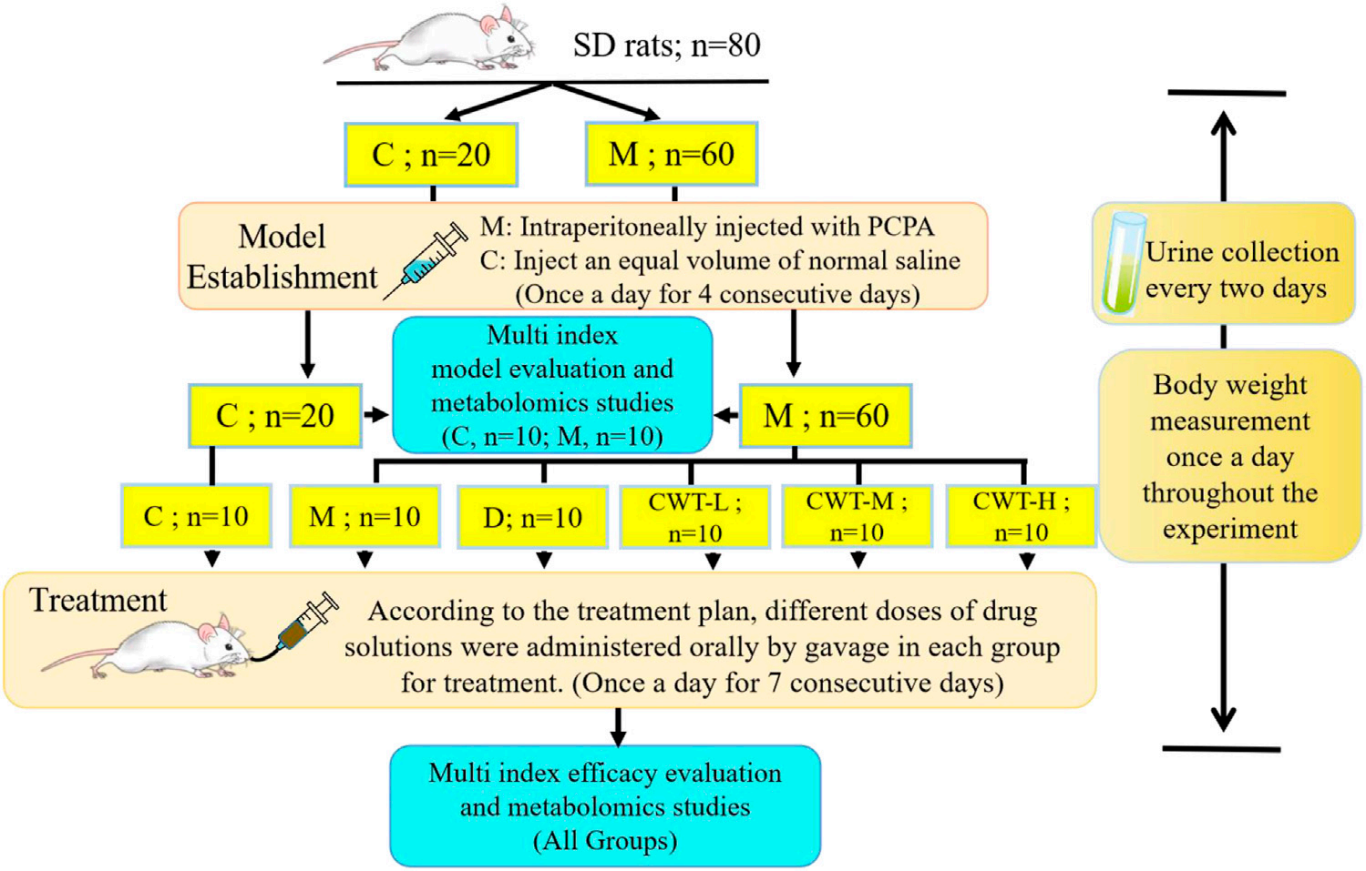
Experimental design flow chart of insomnia rat model generation and CWT pharmacological effects evaluation. (SD rats, Sprague-Dawley rats; C, control group; M, Model group; D, Diazepam group; CWT-L, Ciwujia tablet low-dose group; CWT-M, Ciwujia tablet medium-dose group; CWT-H, Ciwujia tablet high-dose group; PCPA, p-chlorophenylalanine; CWT, Ciwujia Tablet).

CWT and diazepam were respectively ground into powder and fully dissolved in distilled water. Rats were provided with different doses of CWT (CWT-L: 154.2 mg/kg, CWT-M: 308.4 mg/kg, CWT-H: 616.8 mg/kg). The CWT dosage taken by rats was based on previous studies, pharmacological experimental methods, and data from Chinese Pharmacopoeia. The body surface area (BSA) conversion was used to determine the final dosage, which ensured the research significance of this dosage design (Xu et al., 2002; Reagan-Shaw et al., 2008; Lee et al., 2012; Wang et al., 2021). Besides, the rats in the D group were provided with diazepam tablets (0.9 mg/kg). During the treatment process, all groups of rats received the same amounts of water and the drugs were administered once a day for seven consecutive days (Hua et al., 2021). A schematic diagram for the animal modeling, mode of drug administration, and subsequent procedures is shown in Figure 1. All experimental procedures were approved by the Animal Protection and Ethics Committee of the Heilongjiang University of Chinese Medicine, and all experiments were carried out following the Declaration of Helsinki.

### 2.4 Sample collection and preparation

#### 2.4.1 Collection of brain samples

On the fourth day of the insomnia model, the brain tissue of ten rats in the C group and ten rats in the M group was collected. After 7 days of continuous treatment, the brain tissue of all groups was collected and kept in a freezer at −80°C. Testing was carried out in strict accordance with the operating instructions of the ELISA kit.

#### 2.4.2 Collection and preparation of urine samples

The urine of rats in the C and M groups was collected on Day 0, 2, and 4 during insomnia model establishment. Additionally, the urine of rats from all groups was collected on the 1st, 3rd, 5th, and 7th days during the treatment process. The rats were placed in a metabolic cage at 8:00 p.m. on the day of sample collection and the urine was collected until 8:00 a.m. the following day. The urine samples of every rat were prepared by first diluting 1 ml urine with 3 ml distilled water. Next, the mixture was subjected to vortex oscillation for 1 min, followed by centrifugation for 15 min at 4°C and 13,000 r/min. Subsequently, the supernatant was extracted and filtered with a 0.22 μm filter membrane for UPLC-MS detection.

### 2.5 Evaluation of the model and pharmacological effects based on behavioral, energy metabolism, and metabonomics

#### 2.5.1 Daily activities and weight changes

The daily activity patterns of all rats in each group were observed throughout the whole experimental process, including sleep, emotional changes, body hair, and behavior. The weights of the rats in each group were recorded daily to observe the weight change patterns.

#### 2.5.2 Detection of neurotransmitters in the brain

The neurotransmitter content in the brains of rats in the C and M groups was measured on the fourth day of model replication, and in all groups on the final day of treatment. The monoamine neurotransmitters that were measured included 5-hydroxytryptamine (5-HT; Monti, 2011), *γ*-aminobutyric acid (GABA, Kim et al., 2019), dopamine (DA; Dauvilliers et al., 2015), and noradrenaline (NE; Broese et al., 2012). These neurotransmitters are closely related to sleep, emotion, and behavior.

#### 2.5.3 Energy metabolism detection

Various energy metabolism indexes of rats in the C and M groups were detected on the final day of insomnia model establishment, and in all groups on the final day of treatment. These indexes included food intake, water intake (Zuraikat et al., 2021), oxygen consumption rate (Chapman et al., 2018), water exhalation rate, respiratory entropy, and energy consumption rate (de Jonge et al., 2012; Zhang et al., 2021).

#### 2.5.4 Behavioral experiment—open field test

This experiment was performed on the rats under quiet and uniform lighting conditions in the C and M groups on Day 4 of model establishment and the rats in all groups on Day 7 of treatment. An open field box with a length, width, and height of 100 cm × 100 cm × 40 cm was used. The bottom surface of the box was divided into 25 squares, each with an equal area of 20 cm × 20 cm. The bottom and side walls of the box were black. The rats were placed in the center of the open field box for familiarization for 2 min. Besides, the experimental process was filmed with video-recording equipment. The activity of the rats was recorded for 5 min, including the number of squares they passed, the number of times they entered the central area and the residence time, and the number of times they stood up or performed facial grooming. Between each experiment, the inner wall and bottom of the box were cleaned with 75% alcohol, thereby ensuring the accuracy of the experimental results (Bronikowski et al., 2001; Shieh and Yang, 2020).

#### 2.5.5 Metabolic profile assessment

The urine samples collected and prepared in Section 2.4.2 were detected using UPLC-MS and the obtained data were further analyzed by principal components analysis (PCA). Finally, the dynamic changes in the metabolic profile during model establishment and treatment were observed according to the score plot.

### 2.6 Metabonomics research

#### 2.6.1 Analysis conditions of urine biomarkers in UPLC-G2-Si-HDMS

Chromatographic conditions: Chromatographic separation was performed at 35°C with a Waters Acquity UPLC HSS T3 Column (2.1 mm × 100 mm, 1.8 μm) with a mobile phase composed of acetonitrile (A) and water (B), both containing 0.1% v/v formic acid. The elution procedure was performed as follows: 0–8 min, 1%–45% A; 8–10 min, 45%–99% A. The flow rate was maintained at 0.3 ml/min, and the volume of the injected sample was 2 μl.

##### 2.6.1.1 Mass spectrometry conditions

Electrospray ionization (ESI) was employed in this section. In the positive-ion mode, the capillary voltage was 3.0 kV, the cone voltage was 30 V, the source temperature was 110°C, the desolvation gas flow was 600 L/h, and the counterblow air flow rate of the conical hole was 50 L/h. In the negative-ion mode, the capillary voltage was 2.8 kV and the cone voltage was 40 V. The other parameters were the same as those in the positive-ion mode. Additionally, the data acquisition interval was 0.2 s and the scan delay was 0.1 s. Quality data were collected within the range of 50–1,200 Da. Leucine enkephalin (4 ng/ml) was used for quality correction to ensure the accuracy of the MS analysis results.

#### 2.6.2 Metabonomic data processing and analysis

All mass spectrum data files were preprocessed using Progenesis QI, which was composed of chromatographic peak extraction, chromatographic peak alignment, and data normalization. The processed data were imported into Ezinfo for multivariate data analysis. Also, PCA and OPLS-DA were used to construct the score plot. To identify potential biomarkers, different variables among the data of each group were located according to the conditions of VIP > 1, max fold change ≥2, and ANOVA *p*-value ≤ 0.05. Metabolites were determined using the HMDB (http://www.hmdb.ca/), METLIN (http://metlin.scripps.edu/), and KEGG (http://www.kegg.com) databases. Statistical data analysis was performed with the assistance of GraphPad Prism 9 software, and the differences between groups were calculated by the *t*-test or one-way ANOVA. Metabo Analyst (https://www.metaboanalyst.ca/) was employed to conduct pathway analysis.

## 3 Results

### 3.1 Model evaluation based on behavior, energy metabolism, and urine metabolomics

**FIGURE 2:**
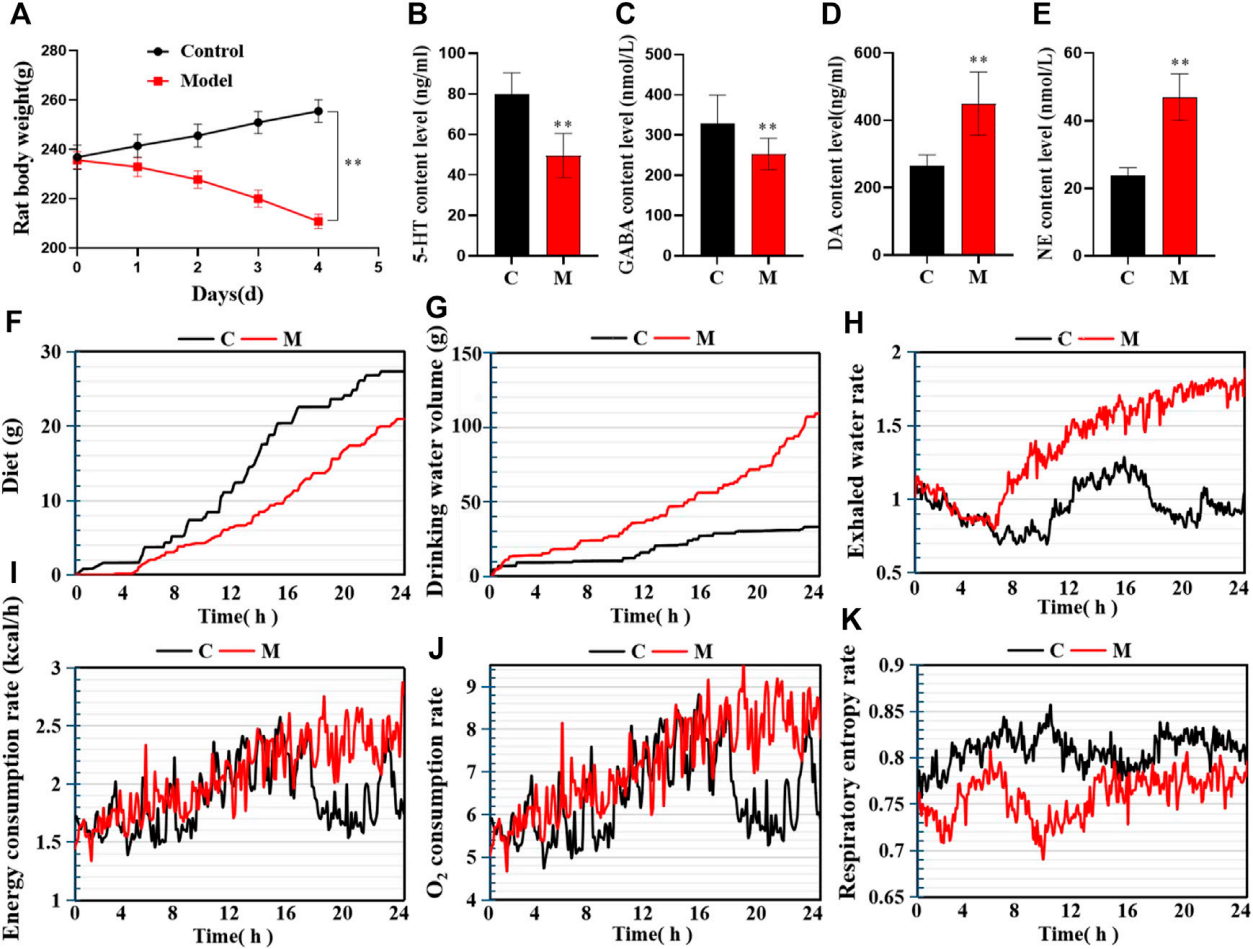
Model evaluation (C, control group, *n* = 10; M, model group, *n* = 10; compared with the C group, *p < 0.05, **p < 0.01, mean ± SD). **(A)** Body weight changes during model establishment; **(B)** 5-HT content; **(C)** GABA content; **(D)** NE content; **(E)** DA content; **(F)** Food intake; **(G)** Water consumption; **(H)** Water exhalation rate; **(I)** Energy consumption; **(J)** Oxygen consumption; **(K)** Respiratory entropy.

In contrast to the C group, 24–30 h after the first intraperitoneal injection of PCPA, the M group rats began to exhibit increased daytime activity. Besides, they were abnormally sensitive to sound and were more prone to gather together, scream, and fight. Thus, the overall observations in the M group differed significantly from those in the C group. By the final day of model establishment, compared to the C group, the rats in the M group were more irritable. Additionally, their fur was fluffy, withered, yellow, and dull, and their circadian rhythms had diminished substantially. Besides, there was a significant difference in body weight between the two groups (*p* < 0.05; Figure 2A). Regarding biochemical indexes, the content of inhibitory neurotransmitters (5-HT and GABA) in the brains of the M group rats had decreased, while that of excitatory neurotransmitters (DA and NE) had increased (*p* < 0.05; Figures 2B–E). On Day 4 of model establishment, the energy metabolism test was performed on the rats. Results revealed that the food intake of rats in the M group had decreased significantly, while the amount of water consumption had increased by about 4 times. Also, the energy consumption rate, water exhalation rate, and oxygen consumption rate were all significantly higher (*p* < 0.05). Furthermore, there was a significant difference in the overall energy metabolism level between the C and M groups (Figures 2F–K).

**FIGURE 3:**
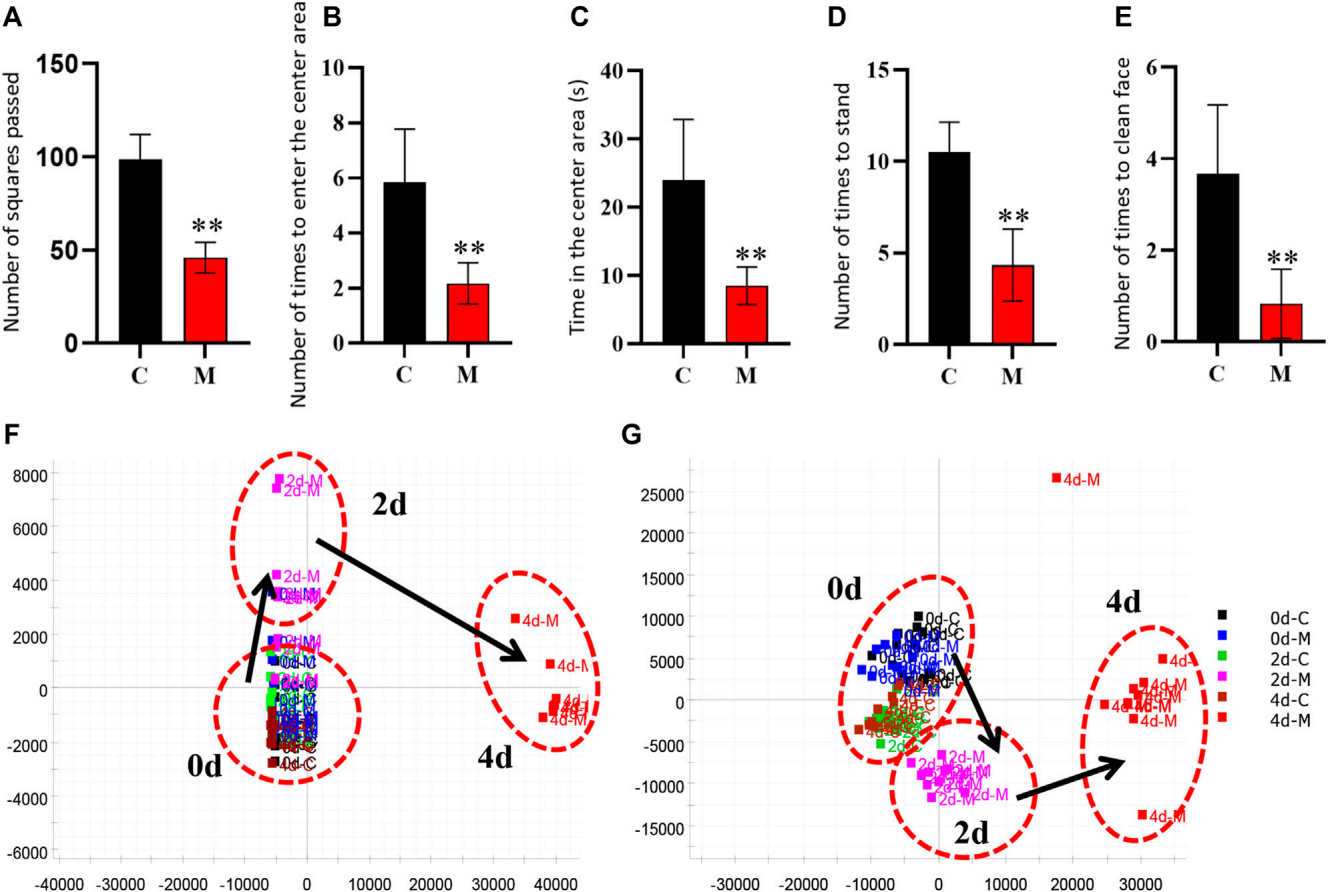
Model evaluation (C, control group, *n* = 10; M, model group, *n* = 10; compared with the C group, *p < 0.05, **p < 0.01, mean ± SD). **(A)** Total number of squares passed; **(B)** Number of times entering the central area; **(C)** Residence time in the central area; **(D)** Total number of times standing; **(E)** Number of instances of facial grooming; **(F)** Score plot of the urine metabolic profile in the positive-ion mode; **(G)** Score plot of the urine metabolic profile in the negative-ion mode.

Similarly, the open field test was performed on the rats in the C and M groups on the final day of insomnia model establishment. The results revealed that exploratory behaviors such as walking and standing were lower in the M group, while autonomous activities were also lower in this group. Besides, the rats in the M group were more sluggish and stayed in the corners of the open field box for a longer time. There were also significant differences in various behavioral indexes between the C and M groups (Figures 3A–E). During the replication of the insomnia model, the score plot of the urine metabolic profile showed that the data of the urine samples in the C group were densely distributed on Day 0, 2, and 4. This indicated that the metabolic profile was stable and exhibited no significant change. However, the metabolic profile of the M group began to deviate on the second day and had diverged more by the fourth day. This indicated that the metabolic network in the M group was seriously disturbed (Figures 3F,G).

The above experimental results revealed that the inhibitory neurotransmitter (5-HT and GABA) content in the brains of insomniac rats decreased, while that of the excitatory neurotransmitters (DA and NE) increased. Additionally, the clinical symptoms of patients with insomnia, such as sleep loss, weight loss, anorexia, dislike of activity, and significant metabolic network disorder, were also observed. This confirmed that the insomnia model was successfully established through the intraperitoneal injection of PCPA.

### 3.2 Biomarker discovery of the insomnia model

**FIGURE 4:**
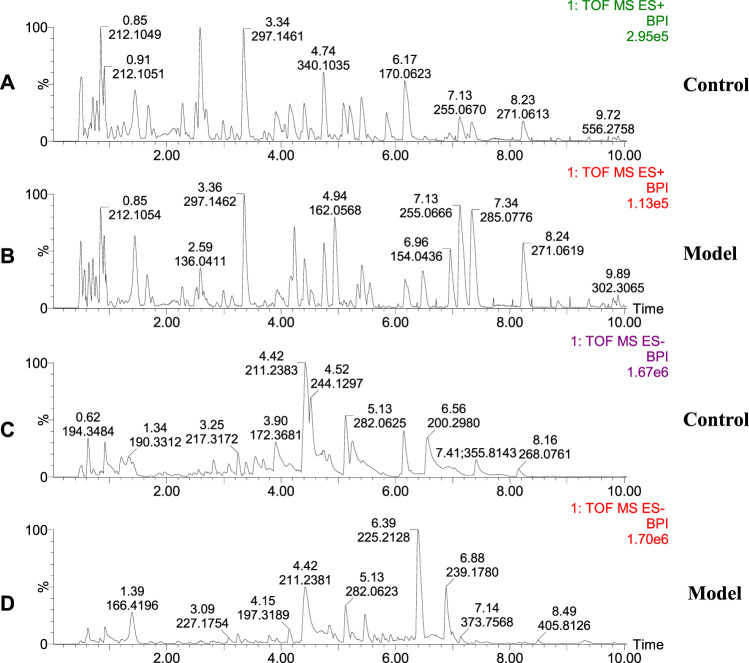
Representative base peak intensity (BPI) of rat urine in groups C and M. **(A)** Urine of group C rats, positive ion mode; **(B)** Urine of group M rats, positive ion mode; **(C)** Urine of group C rats, negative ion mode; **(D)** Urine of group M rats, negative ion mode.

**FIGURE 5:**
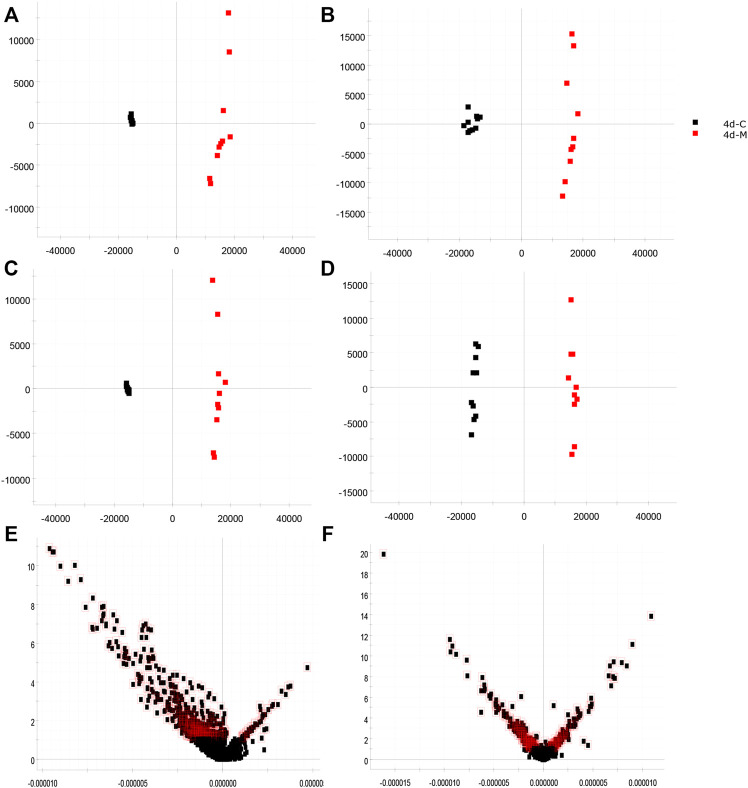
Identification of biomarkers from the insomnia model (red indicates the M group, *n* = 10; black indicates the C group, *n* = 10; red outline indicates VIP > 1). **(A)** PCA in the positive-ion mode; **(B)** PCA in the negative-ion mode; **(C)** OPLS-DA analysis in the positive-ion mode; **(D)** OPLS-DA analysis in the negative-ion mode; **(E)** VIP plot diagram in the positive-ion mode; **(F)** VIP plot diagram in the negative-ion mode.

The urinary metabolites of rats in the C group and M group were analyzed after model establishment according to Section 2.6.1 and Section 2.6.2. Besides, the UPLC-MS detection diagram for the rat urine samples is presented in Figure 4. The PCA and OPLS-DA results indicated that the C group could be differentiated from the M group (Figures 5A–D). This suggested that there was a significant change in the endogenous metabolic network of model rats. Additionally, further analysis was performed to obtain the VIP plot, which directly reflected the information on key factors related to changes in the metabolic profile (Figures 5E,F). The key conditions were identified as VIP > 1, max fold change ≥2, and ANOVA *p*-value ≤ 0.05, while matching was performed using the Human Metabolome Database (HMDB). Ultimately, a total of 28 potential endogenous biomarkers related to insomnia were identified. The detailed identification process and information regarding the urine biomarkers are shown in Supplementary Table S1. The results are listed in Table 1, while the relative content analysis is presented in Supplementary Figure S1.

**TABLE 1:** Identification results and information of urine biomarkers in insomniac rats.

No.	Rt min	M/Z determined	M/Z calculated	Ion species	Scan mode	Proposed composition	Postulated identity	Change trend
1	0.72	168.0633	145.1629	[M + Na]^+^	ESI+	C_6_H_11_NO_3_	Allysine	↑
2	0.85	252.0502	207.1825	[M + FA-H]^-^	ESI-	C_10_H_9_NO_4_	4-(2-Aminophenyl)-2,4-dioxobutanoic acid	↓
3	0.87	259.0939	129.1153	[2M + H]^+^	ESI+	C_5_H_7_NO_3_	1-Pyrroline-4-hydroxy-2-carboxylate	↓
4	0.94	256.1051	255.2314	[M + H]^+^	ESI+	C_9_H_13_N_5_O_4_	7,8-Dihydroneopterin	↑
5	0.96	287.0662	242.1728	[M + FA-H]^-^	ESI-	C_6_H_15_N_2_O_6_P	5-phosphonooxy-L-lysine	↓
6	1.78	259.1661	129.1673	[2M + H]^+^	ESI+	C_6_H_11_NO_2_	Pipecolic acid	↓
7	1.79	263.1029	218.2538	[M + FA-H]^-^	ESI-	C_12_H_14_N_2_O_2_	N-Acetylserotonin	↑
8	2.08	240.1105	239.2304	[M + H]^+^	ESI+	C_9_H_13_N_5_O_3_	Dihydrobiopterin	↑
9	2.20	207.0631	184.1902	[M + Na]^+^	ESI+	C_9_H_12_O_4_	Vanylglycol	↓
10	2.83	177.0567	176.2148	[M + H]^+^	ESI+	C_10_H_12_N_2_O	Serotonin	↓
11	3.41	137.0601	136.1504	[M + H]^+^	ESI+	C_8_H_8_O_2_	4-Hydroxyphenylacetaldehyde	↓
12	3.68	261.1317	130.1308	[2M + H]^+^	ESI+	C_6_H_10_O_3_	Ketoleucine	↑
13	3.76	160.0778	137.1182	[M + Na]^+^	ESI+	C_8_H_11_NO	Tyramine	↓
14	4.03	198.1136	197.2338	[M + H]^+^	ESI+	C_10_H_15_NO_3_	Metanephrine	↓
15	4.20	202.0467	179.1705	[M + Na]^+^	ESI+	C_9_H_9_NO_3_	Hippuric acid	↑
16	4.22	307.1663	153.1803	[2M + H]^+^	ESI+	C_8_H_11_NO_2_	Dopamine	↓
17	4.66	377.1463	376.4024	[M + H]^+^	ESI+	C_17_H_20_N_4_O_6_	Riboflavin	↓
18	4.75	301.2132	278.4107	[M + Na]^+^	ESI+	C_18_H_30_O_2_	Alpha-Linolenic acid	↓
19	4.81	175.1239	174.2416	[M + H]^+^	ESI+	C_11_H_14_N_2_	N-Methyltryptamine	↓
20	5.42	293.1470	146.1407	[2M + H]^+^	ESI+	C_5_H_10_N_2_O_3_	L-Glutamine	↑
21	5.58	323.1464	161.1638	[2M + H]^+^	ESI+	C_6_H_11_NO_4_	Aminoadipic acid	↑
22	5.82	249.1236	248.2849	[M + H]^+^	ESI+	C_13_H1_6_N_2_O_3_	6-Hydroxymelatonin	↑
23	6.13	241.1700	121.1862	[2M-H]^-^	ESI-	C_8_H_11_N	1-Phenylethylamine	↑
24	6.17	144.0778	121.1862	[M + Na]^+^	ESI+	C_8_H_11_N	Phenylethylamine	↑
25	6.46	227.2026	228.3716	[M + H]^+^	ESI-	C_14_H_28_O_2_	Myristic acid	↑
26	7.00	269.0790	224.2104	[M + FA-H]^-^	ESI-	C_10_H_12_N_2_O_4_	L-3-Hydroxykynurenine	↑
27	7.34	286.1145	241.2531	[M + FA-H]^-^	ESI-	C_9_H_15_N_5_O_3_	Tetrahydrobiopterin	↑
28	9.78	277.2173	276.4016	[M + H]^+^	ESI+	C_18_H_28_O_2_	Stearidonic acid	↑

Note: ↑ or ↓ represents an increase or decrease of biomarkers in the urine of Group M rats compared with the Group C rats.

### 3.3 Evaluation of pharmacological effects based on behavior, energy metabolism, and urine metabonomics

**FIGURE 6:**
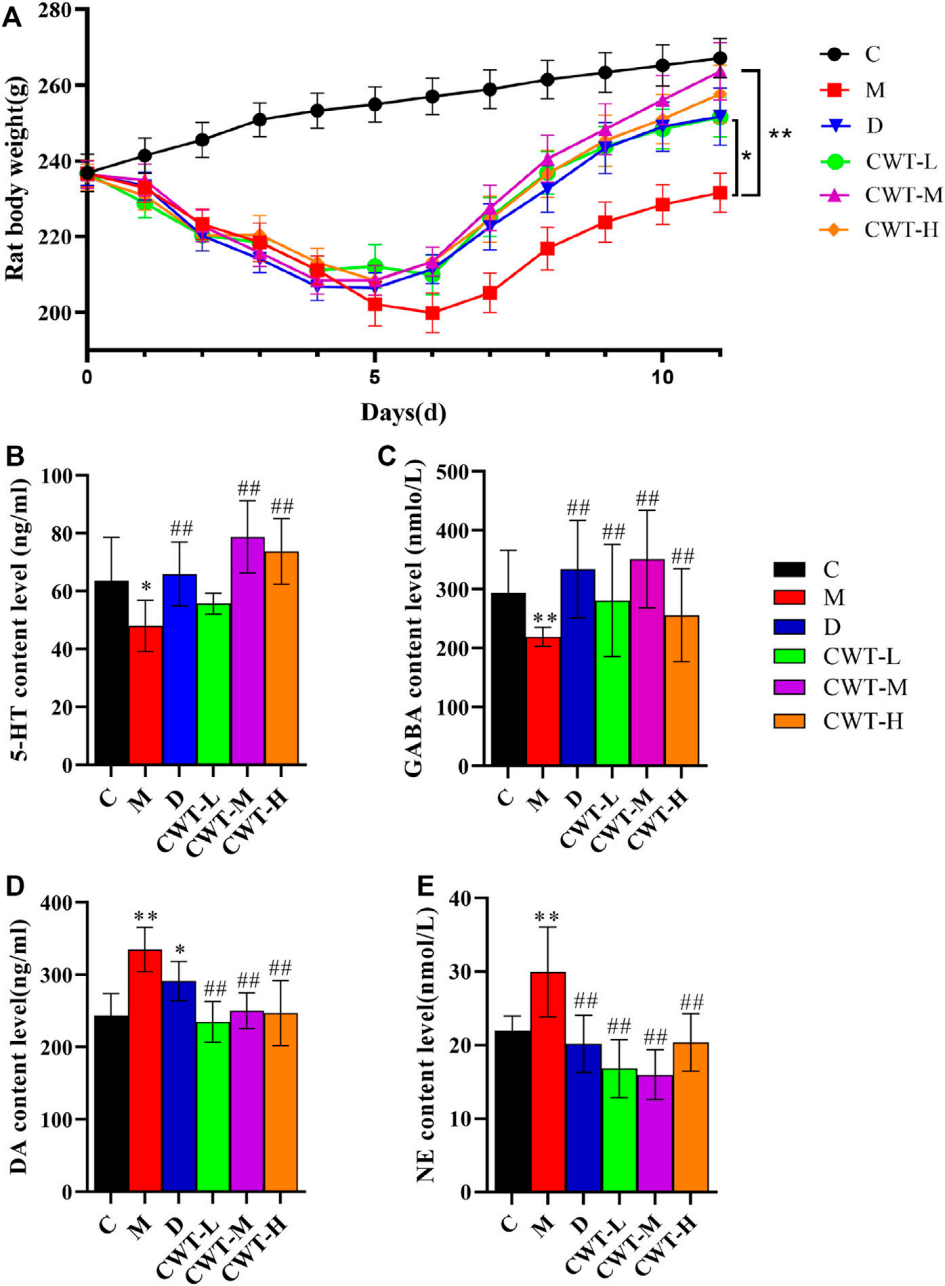
Body weight and biochemical evaluation (C, control group, *n* = 10; M, model group, *n* = 10; D: diazepam group, *n* = 10; CWT-L group, *n* = 10; CWT-M group, *n* = 10; CWT-H group, *n* = 10; compared with the C group, *p < 0.05, **p < 0.01; compared with the M group, ^#^ *p* < 0.05, ^##^ *p* < 0.01, mean ± SD). **(A)** Body weight changes in each group during treatment; **(B)** Analysis of 5-HT content in the brains of rats; **(C)** Analysis of GABA content; **(D)** Analysis of DA content; **(E)** Analysis of NE content.

The rats in each group were treated for 7 days according to the administration method described in Section 2.3.2. After treatment, the experiments were conducted again according to the indexes evaluated in the insomnia model (Figure 6A).

**FIGURE 7:**
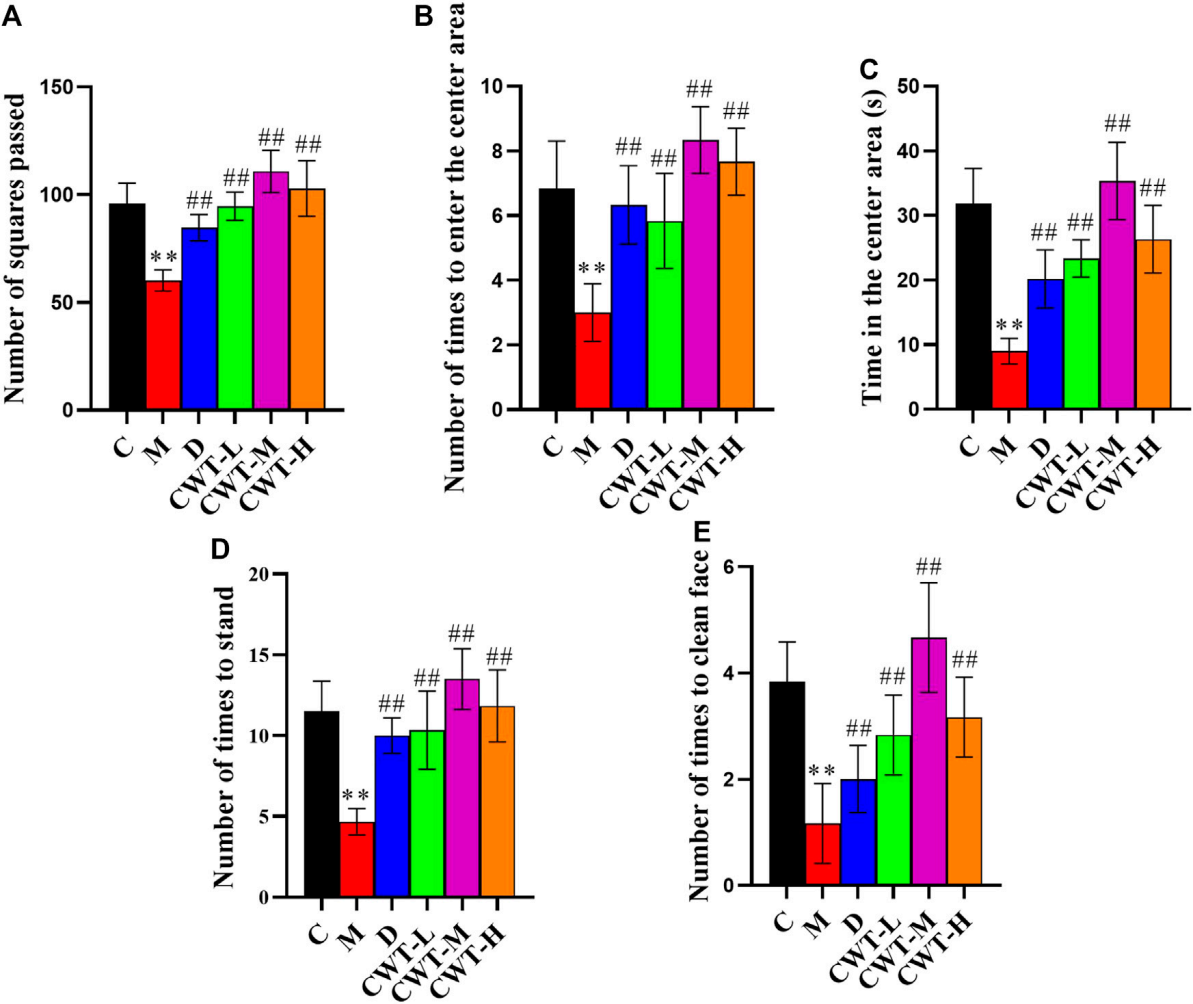
Behavioral analysis results for the rats in each group after 7 days of treatment (C, control group, *n* = 10; M, model group, *n* = 10; D, diazepam group, *n* = 10; CWT-L group, *n* = 10; CWT-M group, *n* = 10; CWT-H group, *n* = 10; compared with the C group, *p < 0.05, **p < 0.01; compared with the M group, ^#^ *p* < 0.05, ^##^ *p* < 0.01, mean ± SD). **(A)** Total number of squares passed; **(B)** Number of times the rats entered the central area; **(C)** Residence time in the central area; **(D)** Standing times; **(E)** Instances of facial grooming.

We discovered that the body weight of the rats in the D group and the CWT treatment group began to recover on the second day of treatment. Furthermore, their body weight almost matched that of the rats in Group C by the final day of treatment, and the CWT-M group exhibited the best recovery effect. Although the body weight of the rats in the M group recovered to a certain extent during the experiment, there was still a significant difference in body weight between Group C and Group M at the end of the experiment. The neurotransmitter detection results showed that after 7 days of continuous treatment, the content of 5-HT, GABA, DA, and NE in the brains of rats in the D group and the CWT treatment group essentially recovered to the same level as in the C group. However, there was still a considerable difference between the M group and the C group (Figures 6B-E). The results of the open field test showed that compared to the M group, the rats in the D group and the CWT treatment groups had more autonomous behavior activity, standing, and facial grooming behavior. There was also a tendency to return to the normal levels of the Group C rats (Figure 7).

**FIGURE 8:**
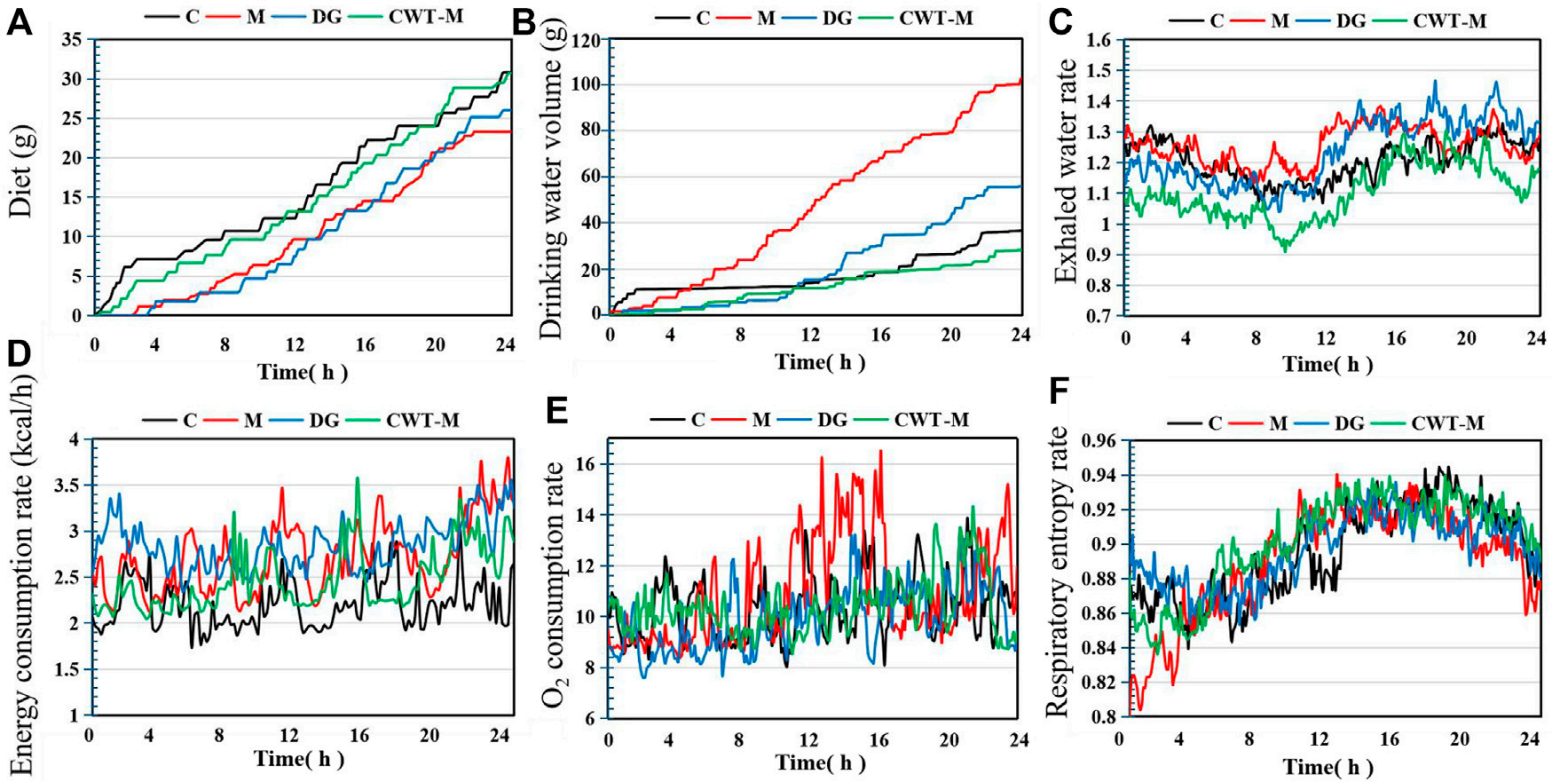
Basic metabolic evaluation of rats in each group on Day 7 of treatment (C, control group, *n* = 10; M, model group, *n* = 10; DG, diazepam treatment group, *n* = 10; CWT-M group, *n* = 10). **(A)** Food intake of rats in each group; **(B)** Water consumption; **(C)** Water exhalation rate; **(D)** Energy consumption rate; **(E)** Oxygen consumption rate; **(F)** Respiratory entropy.

These results demonstrated that compared with the D group, the recovery effect of the CWT treatment groups was more obvious. Among them, the CWT-M group achieved the best recovery effect. Therefore, the CWT-M group, C group, M group, and D group were selected for subsequent energy metabolism experiments (Figure 8).

The experimental results regarding energy metabolism indicated that the rats in the M group still showed a higher level of water consumption and a lower level of food intake. Besides, the energy consumption rate, oxygen consumption rate, and water exhalation rate in the M group were all substantially higher than those in the C group. However, the food intake, water consumption, water exhalation rate, oxygen consumption rate, energy consumption rate, and respiratory entropy of the rats in the D and CWT-M groups generally returned to the normal levels of rats in the C group. Compared to the D group, the recovery effect of the CWT-M group was more significant.

**FIGURE 9:**
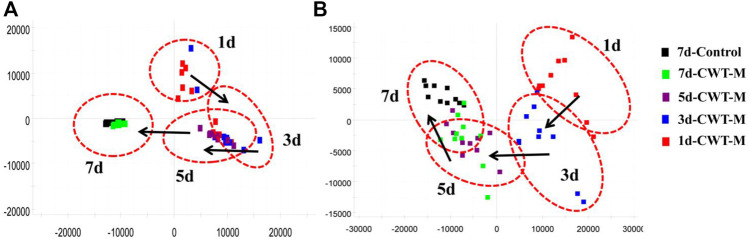
PCA analysis diagram of dynamic changes in the urine metabolic profile in the CWT-M group during 7 days of continuous treatment. **(A)** PCA analysis of the urine metabolic profile in the positive-ion mode; **(B)** PCA analysis of the urine metabolic profile in the negative-ion mode.

**FIGURE 10:**
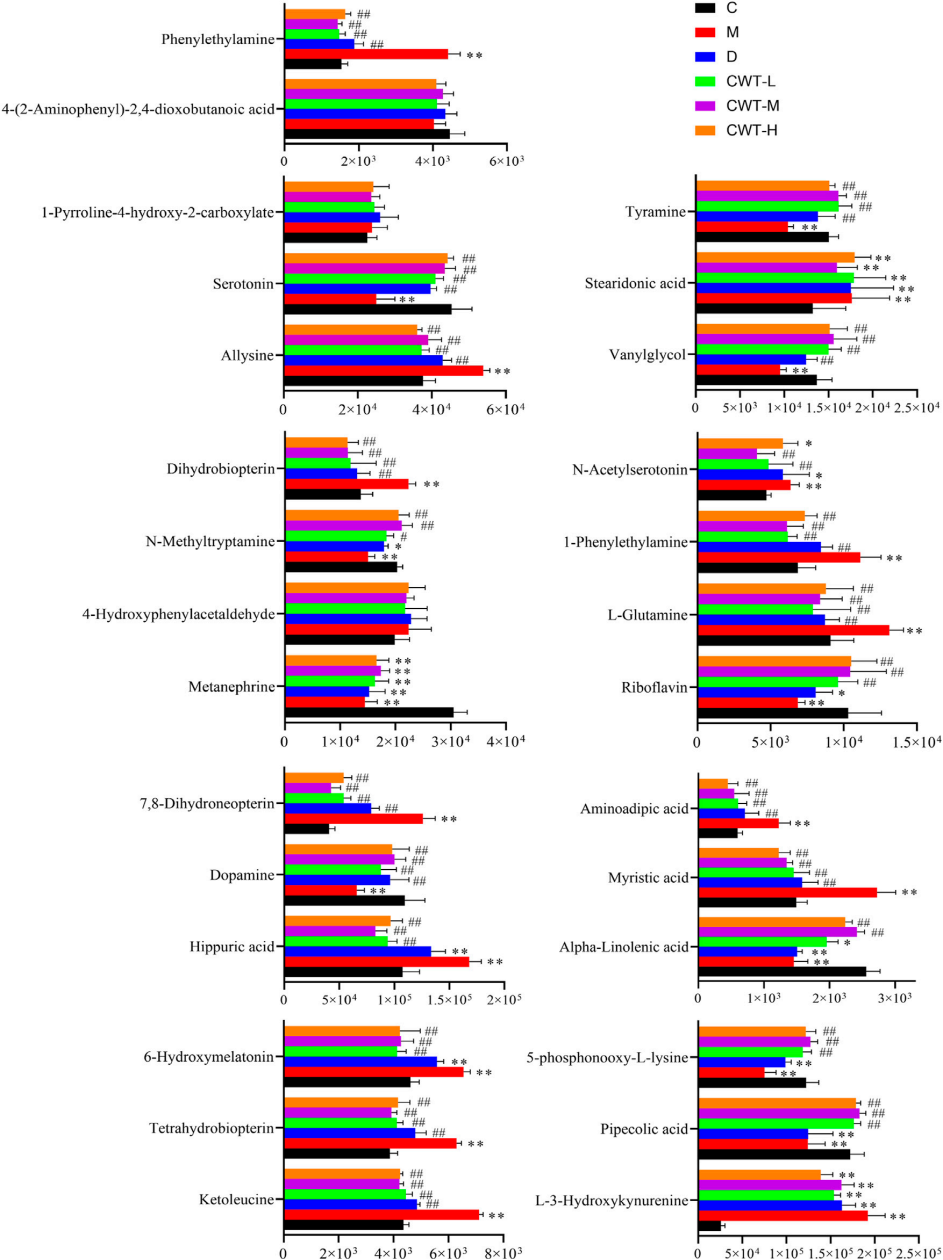
Changes in the content of insomnia biomarkers in each group after treatment. (C, control group, *n* = 10; M, model group, *n* = 10; D, diazepam group, *n* = 10; CWT-L group, *n* = 10; CWT-M group, *n* = 10; CWT-H group, *n* = 10; compared with the C group, *p < 0.05, **p < 0.01; compared with the M group, #*p* < 0.05, ##*p* < 0.01, mean ± SD).

The urine samples of rats in each group were analyzed during treatment and the PAC analysis was performed on the urine sample data of the CWT-M group to observe the effect of CWT on the *in vivo* metabolic profile during treatment. We discovered that the metabolic spectrum of rats in the CWT-M group tended to be far away from that in the M group but close to that in the C group during treatment (Figure 9). Besides, changes in the content of urinary biomarkers in each group were analyzed after 7 days of treatment. Among them, 21 biomarkers in the CWT-L group, 22 biomarkers in the CWT-M group, and 21 biomarkers in the CWT-H group returned to normal levels (*p* < 0.05). In contrast, only 14 biomarkers in the D group returned to typical levels (*p* < 0.05). Taking the raw abundance of biomarkers as an indicator, the relative content change and recovery status of the biomarkers for rats in each group were analyzed. Detailed information concerning the relative content and recovery status of the biomarkers are shown in Supplementary Tables S2, S3, while the analysis results are illustrated in Figure 10.

### 3.4 Target and metabolic pathway analysis

**FIGURE 11:**
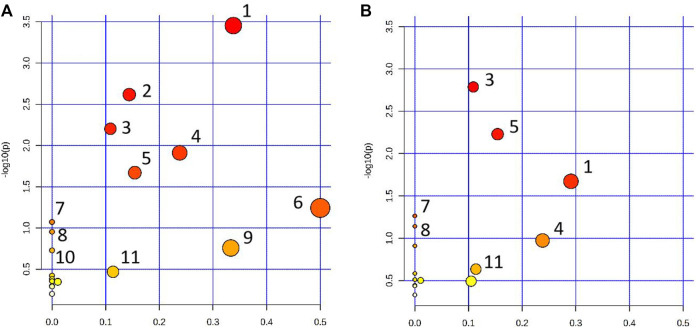
Metabolic pathway analysis results. **(A)** Pathway analysis of rat urine samples in the CWT treatment group; **(B)** Pathway analysis of rat urine samples in the D group (1: Lysine degradation; 2: Tryptophan metabolism; 3: Folate biosynthesis; 4: Phenylalanine metabolism; 5: Tyrosine metabolism; 6: Riboflavin metabolism; 7: Nitrogen metabolism; 8: Valine, leucine and isoleucine biosynthesis; 9: alpha-Linolenic acid metabolism; 10: Arginine biosynthesis; 11: Alanine, aspartate, and glutamate metabolism).

The pathway analysis was performed on 22 urine biomarkers in the CWT treatment group through the Metabo Analyst (https://www.metaboanalyst.ca/) website. Thus, the metabolic pathway of CWT in the treatment of insomnia was identified, including lysine degradation, tryptophan metabolism, folate biosynthesis, phenylalanine metabolism, tyrosine metabolism, riboflavin metabolism, D-glutamine and D-glutamate metabolism, nitrogen metabolism, and valine, leucine, and isoleucine biosynthesis (Figure 11A; Supplementary Table S4). For comparison, the metabolic pathways involving 14 insomnia urine biomarkers in the D group were also analyzed (Figure 11B; Supplementary Table S5). The comparative analysis was carried out according to the pathways and biomarkers regulated by CTW and diazepam, and the results are shown in Table 2.

**TABLE 2:** Comparison of biomarker quantity and metabolic pathway affected by CWT and diazepam.

No.	Pathway name	Biomarker matching quantity	
		CWT	Diazepam
1	Lysine degradation	4	2
2	Tryptophan metabolism	4	1
3	Folate biosynthesis	3	3
4	Phenylalanine metabolism	2	1
5	Tyrosine metabolism	3	3
6	Riboflavin metabolism	1	0
7	D-Glutamine and D-glutamate metabolism	1	1
8	Nitrogen metabolism	1	1
9	Valine, leucine, and isoleucine biosynthesis	1	1
10	alpha-Linolenic acid metabolism	1	0
11	Arginine biosynthesis	1	1
12	Alanine, aspartate, and glutamate metabolism	1	1
13	Glyoxylate and dicarboxylate metabolism	1	1
14	Biosynthesis of unsaturated fatty acids	1	0
15	Pyrimidine metabolism	1	1
16	Valine, leucine, and isoleucine degradation	1	1
17	Fatty acid biosynthesis	1	1
18	Aminoacyl-tRNA biosynthesis	1	1
19	Purine metabolism	1	1

**FIGURE 12:**
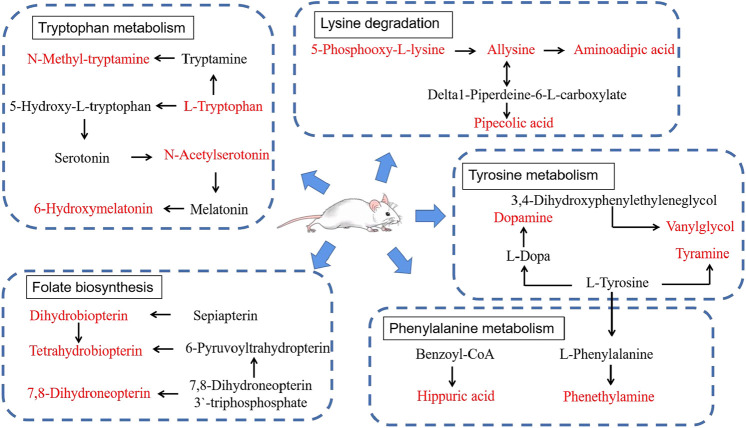
Main correlation network of potential biomarkers that CWT significantly reverses (red font represents potential biomarkers).

According to the above experimental results concerning behavior, energy metabolism, and metabolomics, CWT had an excellent effect on the insomnia model of rats. This effect was mainly achieved by regulating lysine degradation, tryptophan metabolism, folate biosynthesis, phenylalanine metabolism, tyrosine metabolism, riboflavin metabolism, D-glutamine and D-glutamate metabolism, nitrogen metabolism, valine, leucine, and isoleucine biosynthesis, and the metabolism pathway. After treatment, their expression returned to normal levels, thereby playing a role in the treatment of insomnia. Based on the -log(*p*) values of the pathway analysis results in the above-mentioned CWT-treated insomnia rats, the top five metabolic pathway networks were mapped to clarify the potential mechanism (Figure 12).

## 4 Discussion

Insomnia is a condition that exerts a profound impact on the emotions, behavior, and physical state of people in daily life. Anxiety and depression are common complications of this disease, which may induce anorexia (Allison et al., 2016), irritability, fatigue, reduced behavioral activity, and other symptoms. In this study, an animal model was established based on the clinical symptoms of patients with insomnia. As an inhibitor for the neurotransmitter serotonin synthase, p-chlorophenylalanine (PCPA) was selected in this experiment to inhibit the synthesis of 5-HT in the brains of rats. This led to the disappearance of circadian rhythms, thereby resulting in insomnia in healthy rats. This model featured a short preparation period and strong repeatability. As a result, it has become a widely accepted insomnia model both domestically and internationally.

Based on behavior, energy metabolism, and metabolomics research, the insomnia model was evaluated. After model establishment, the pharmacological effects of CWT in the treatment of insomnia were evaluated. Besides, metabolomics was explored to ensure the reliability and accuracy of the experimental results. Additionally, the stability and precision of the UPLC-MS system were investigated, achieving satisfactory results.

Compared with diazepam tablets, CWT achieved a superior curative effect on insomnia. CWT increases the content of inhibitory neurotransmitters (5-HT and GABA) in the brain and reduces the content of excitatory neurotransmitters (DA and NE). It also increases the activity of independent exploration, stabilizes emotions, contributes to sleep, and prevents the stimulation of everyday emotions. Additionally, after CWT treatment, the basic metabolic indicators (such as respiratory entropy, energy consumption rate, water exhalation rate, and O_2_ consumption) returned to normal levels and were maintained at a stable state. Moreover, the basic daily metabolic disorders associated with insomnia also returned to a normal state. Also, CWT more effectively regulates lysine degradation and tryptophan metabolism pathways and regulates its unique riboflavin metabolism, alpha-linolenic acid metabolism, and unsaturated fatty acid biosynthesis metabolism pathways during treatment. It has been demonstrated in some clinical studies that riboflavin effectively inhibits frailty syndromes including sleep disorders (Esipov et al., 2019), alleviates insomnia, anxiety, and stress, and improves the quality of life of patients (Abbaszadeh et al., 2021). Alpha-linolenic acid is also commonly used in the treatment of nervous system diseases, such as insomnia, cognitive impairment, and paralysis (Aleem, 2021). The production and intake of unsaturated fatty acids also have a specific impact on insomnia (Cheng et al., 2016; Dai and Liu, 2021). These results demonstrate that CWT has more clinical advantages in the treatment of insomnia.

The target and pathway analysis results revealed that among the 22 endogenous biomarkers of insomnia regulated by CWT, serotonin (5-HT), 6-hydroxymelatonin, N-acetylserotonin, and N-methyltryptamine were involved in tryptophan metabolism. According to relevant research, tryptophan metabolism is closely related to sleep (Malhotra et al., 2017; van Dalfsen and Markus, 2019), emotions (Jenkins et al., 2016), and behavior (Young, 2013; Steenbergen et al., 2016). Therefore, based on these experimental results, the effect of CWT on the tryptophan metabolic pathway in the treatment of insomnia was also explored.

Tryptophan (Trp) is an essential amino acid and an important part of the human diet. Several studies suggest that this amino acid supplement can be used in the treatment of depression and sleep disorders since tryptophan is related to the synthesis of serotonin (5-HT) and melatonin (Kałużna-Czaplińska et al., 2019). Serotonin (5-HT) is an indoleamine compound that serves as a monoamine neurotransmitter, biochemical messenger, and regulator in mammals (Berger et al., 2009). Besides, it plays a role in the central nervous system (CNS) and the peripheral nervous system (Grimaldi and Fillion, 2000). As an essential amino acid, L-tryptophan is hydroxylated by tryptophan hydroxylase to form 5-hydroxytryptophan and then decarboxylated by L-tryptophan decarboxylase to form 5-HT. In the nervous system, 5-HT serves as an inhibitory neurotransmitter in the synapse, and is closely related to daily behavior, emotions, and “sleep-wake”. Besides, according to some studies, 5-HT systematically promotes the sleep of zebrafish and rodents at night (Oikonomou et al., 2019).

Results from the analysis of neurotransmitter content in our experiments indicated that the content of 5-HT in the brains of insomniac rats was significantly lower than that of normal rats. This result may be caused by the decrease in the activity of L-tryptophan decarboxylase, which synthesizes 5-HT *in vivo* and induces a decrease in 5-HT synthesis. Meanwhile, 5-HT is continuously converted and consumed by N-acetyltransferase until it is eventually exhausted, resulting in tryptophan metabolism disorders. The decrease of 5-HT also leads to disorders in the nervous system, which further induces insomnia, anxiety, stereotypical behavior, disappearance of circadian rhythms, and other symptoms in rats.

According to the synthetic and metabolic pathways of the above four biomarkers in the tryptophan pathway and combined with the content of these four biomarkers in insomniac rats before and after treatment, it can be inferred that the curative effect of CWT on insomnia may be achieved as follows. First, the effective components *in vivo* act on L-tryptophan decarboxylase, which increases the activity of L-tryptophan decarboxylase, thereby increasing the content of 5-HT and enhancing 5-HT metabolism. The activity of N-acetyltransferase and CYP1A1 in the follow-up pathway increases indirectly and the content of N-acetylserotonin and 6-hydroxymelatonin begins to return to normal levels. Furthermore, the components in the body may also directly act on N-acetyltransferase, N-methyltransferase, and other enzymes to enhance their activity and increase the content of N-acetylserotonin and N-methyltryptamine. Eventually, the tryptophan metabolism in rats returns to normal levels. Besides, the daily behavior, emotions, and circadian rhythm of rats also return to a normal state, thus curing insomnia. This hypothesis can be verified by the recovery of 5-HT content in the brains of insomniac rats after CWT treatment.

It should be noted that CWT regulates 14-3-3ε and 14-3-3ζ (Zhou et al., 2018). Specifically, 14-3-3ε is required for the stabilization of tryptophan hydroxylase (TPH2) and, therefore, the conversion of tryptophan to serotonin. Additionally, 14-3-3ζ is necessary for the stabilization of arylalkylamine N-acetyltransferase (AANAT), the conversion of serotonin to N-acetylserotonin (NAS), and the initiation of the melatonergic pathway (Anderson and Maes, 2014). In future research, it will be important to determine the direct and indirect role of CWT in the regulation of these 14-3-3 isoforms across different cell types, including astrocytes and pinealocytes.

Data suggests that the p-chlorophenylalanine insomnia model is regulated by the traditional Chinese medicine Armillaria mellea (Vahl) P. Kumm. It ameliorates insomnia with effects on both tryptophan metabolism and the gut microbiome (Yao et al., 2022). Future research should clarify the impact of CWT on gut permeability and the gut microbiome, especially the regulation of butyrate production, given the importance of butyrate as an epigenetic regulator and optimizer of mitochondrial function. It will also be valuable to determine the effects of CWT on mitochondrial metabolism, given that acetyl-CoA is a necessary cosubstrate for the initiation of the melatonergic pathway and, therefore, NAS and melatonin production.

## 5 Conclusion

In this study, we established an insomniac rat model with the injection of p-chlorophenylalanine. Besides, the effect of CWT was evaluated based on daily energy metabolism, behavioral tests, brain neurotransmitter levels, and metabonomics methods. In the insomniac rat model, a total of 28 endogenous urine metabolites were identified as potential biomarkers. Among them, 22 biomarkers were regulated by CWT. The metabolic pathways included tryptophan metabolism, lysine degradation, folate biosynthesis, phenylalanine metabolism, tyrosine metabolism, riboflavin metabolism, and so on. These findings provide a more comprehensive insight into the mechanism of CWT in the treatment of insomnia. This crucial mechanism may be achieved by increasing the activity of L-tryptophan decarboxylase, N-acetyltransferase, and CYP1A1, increasing the content of 5-HT, and restoring the expression of N-acetylserotonin, N-methyltryptamine, and 6-hydroxymelatonin to normal levels. Consequently, the metabolic pathway can be restored to a normal and stable state, thereby achieving pharmacological effects in the treatment of insomnia.

## Data Availability

The original contributions presented in the study are included in the article/Supplementary Material, further inquiries can be directed to the corresponding author.

## Abbreviations

CWT, Ciwujia tablets; TCM, Traditional Chinese medicine; SD, Sprague dawley; PCPA, 4-chlorophenylalanine; UPLC-Q-TOF-MS/MS, Ultra performance liquid chromatography-quadrupole-time-of-flight mass spectrometry; PCA, Principal component analysis; VIP, Variable importance in projection; OPLS-DA, Orthogonal projections to latentstructures discriminant analysis; ESI, Electrospray ionization; Rt, Retention time; M/Z, Mass-to-charge ratio; ELISA, Enzyme linked immunosorbent assay; 5-HT, 5-hydroxytryptamine; GABA, γ-aminobutyric acid; DA, Dopamine; NE, Norepinephrine; HMDB, Human metabolome database; KEGG, Kyoto encyclopedia of genes and genomes; NAS, N-acetylserotonin; AANAT, N-acetyltransferase; TPH2, Tryptophan hydroxylase.
