# Supplementary material for: Evaluation of the pharmacological effects and exploration of the mechanism of traditional Chinese medicine preparation Ciwujia tablets in treating insomnia based on ethology, energy metabolism, and urine metabolomic approaches
Source: Front Pharmacol. 2022 Dec 5;13:1009668. doi: 10.3389/fphar.2022.1009668 (PMC9760683; doi:10.3389/fphar.2022.1009668)
Supplement: Supplementary file 6 [file Image1.pdf]

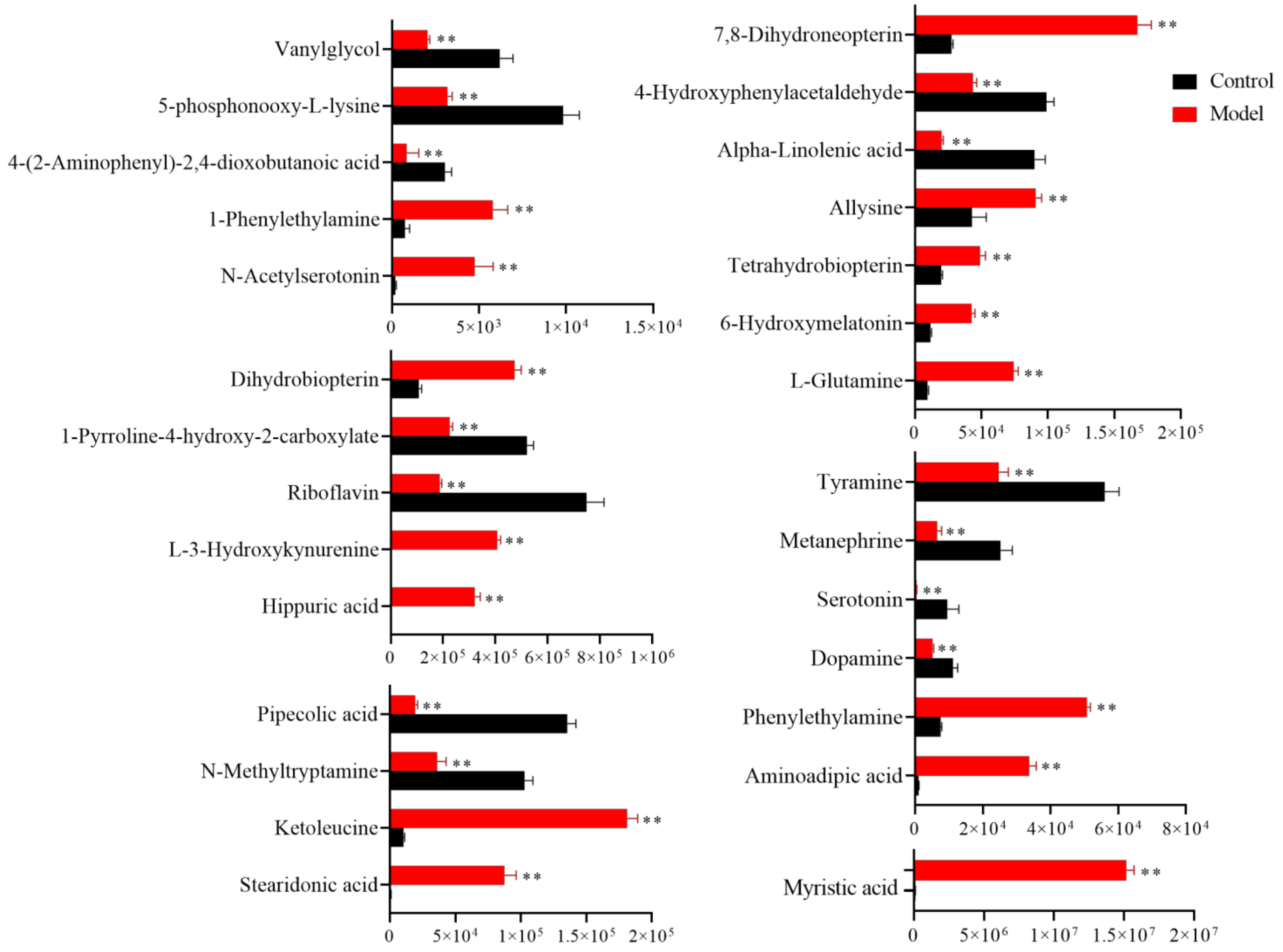

Supplementary Figure 1: Changes in content of insomnia biomarkers in the C and M groups (C: control group, n = 10; M: model group, n = 10; compared with the C group, * $P < 0.05$, ** $P < 0.01$, mean±SD).
